# Framing effects on smoking cessation intentions: A quasi-experimental study of gain- versus loss-framed text messages among male smokers in China

**DOI:** 10.18332/tid/216244

**Published:** 2026-02-17

**Authors:** Jiawei Jin, Yannan Jiang, Wei Guo, Shuhan Jiang

**Affiliations:** 1 School of Humanities and Management, Zhejiang Chinese Medical University, Hangzhou, China; 2 Department of Statistics, Faculty of Science, University of Auckland, Auckland, New Zealand; 3 Hangzhou Hospital for Prevention and Treatment of Occupational Diseases, Hangzhou, China; 4 Department of General Practice and Primary Health Care, School of Population Health, University of Auckland, Auckland, New Zealand

**Keywords:** smoking cessation intention, framing effect, Chinese male smokers, self-efficacy, self-exempt beliefs

## Abstract

**INTRODUCTION:**

China has over 300 million smokers, yet overall willingness to quit remains low. Moreover, more than 90% of Chinese smokers who attempt to quit have no professional support, underscoring the need for effective self-directed cessation interventions.

**METHODS:**

We conducted a pretest–posttest quasi-experimental study in Hangzhou, China (March–June 2023). A total of 1082 eligible adult male smokers were allocated to receive gain-framed (n=546) or loss-framed (n=536) cessation messages after baseline assessments. Quit intention was measured with a 5-point Likert scale immediately before and after exposure to the allocated message. The primary outcome was an increase in quit intention post-exposure. Logistic regression was used to compare framing effects between groups, adjusting for occupation, annual household income, baseline quit intention, self-exempt beliefs, self-efficacy, and nicotine dependence (FTND). Subgroup analyses explored effect modification by these psychological factors.

**RESULTS:**

All participants received the allocated intervention and 1081 were included in the final analysis. Increased quit intention was observed in 34.6% of the gain-framed group versus 30.7% of the loss-framed group (adjusted odds ratio, AOR=0.73; 95% CI: 0.55–0.98; p=0.038). Subgroup analyses showed consistent benefits of gain-framed messages, with stronger associations among participants with low self-exempt beliefs (AOR=0.66; 95% CI: 0.45–0.96; p=0.031) and low self-efficacy (AOR=0.48; 95% CI: 0.28–0.83; p=0.008).

**CONCLUSIONS:**

Gain-framed messages were associated with higher quit intentions than loss-framed messages among Chinese male smokers. Tailoring message framing to smokers’ psychological profiles may yield more favorable responses. Given the quasi-experimental design, further studies are needed to obtain sufficient evidence for culturally sensitive tobacco control strategies in China.

## INTRODUCTION

Smoking is one of the leading causes of preventable deaths worldwide. China is the world’s largest producer and consumer of tobacco products, with more than 300 million smokers and over 1 million deaths each year from tobacco-related diseases^1^. However, the overall willingness to quit smoking among Chinese smokers remains low: a meta-analysis of studies on smoking cessation intentions in China published since 2008 found that only 31.8% of smokers nationwide expressed a desire to quit smoking^2^. According to Ajzen’s Theory of Planned Behavior (TPB)^3^, behavioral intentions are influenced by both behavioral attitudes and subjective norms, and are a direct factor in determining actual behavior. Short message service (SMS)-based interventions have proven to be effective, cost-efficient and widely applicable^4^ with the framing effects theory providing essential theoretical support for crafting effective smoking cessation messages^5^. While new technologies have expanded the delivery channels for smoking cessation interventions, the effectiveness of the message text itself continues to serve as the important foundation for the success of more complex intervention strategies.

The ‘framing effects’ refer to the phenomenon whereby people exhibit different preferences when the same issue is presented in different ways^6^. Salovey et al.^7^ pointed out that gain-framing is more persuasive in promoting preventive behaviors, such as smoking cessation and physical exercise, while loss-framing is more persuasive in promoting early detection behaviors, such as breast self-examination and HIV testing. Particularly regarding smoking warnings, Schneider et al.^8^, who were among the first to identify the framing effects, demonstrated through their research that smokers exposed to gain-framing warnings significantly reduced cigarette consumption in the subsequent month compared with those receiving loss-framing warnings. This conclusion has garnered support from numerous subsequent scholars. For example, research employing combined print materials and videos further confirmed that gain-framing may be more persuasive than loss-framing in promoting smoking cessation behavior change^9^. Similarly, a study on pictorial health warnings on cigarette packs showed that gain-framing on unbranded plain packaging elicited stronger motivation to quit^10^.

However, it is worth noting that when the framing information involves other important factors, differences in effectiveness between different groups begin to emerge. Klein et al.^11^ conducted empirical research on smoking cessation information related to pregnancy among women of childbearing age and found that information focusing on the benefits of quitting smoking was more effective. However, in a study of dual-smoker couples by Lipkus et al.^12^, it was found that loss-framing may be more persuasive than gain-framing. Furthermore, Kim and Lee^13^ pointed out that when exposed to first-person narratives, the stage of smoking cessation that smokers are in also leads to differences in the framing effects: smokers who have not yet considered quitting are more susceptible to the incentives of the loss-framing, while smokers who are considering or preparing to quit are more motivated by the gain-framing. In summary, although most studies support the relative advantage of the gain-framing approach in smoking cessation interventions, the framing effects are influenced by various factors. Different types of smokers respond differently to gain- or loss-framed information, and even contradictory results may arise. Therefore, further research is needed to explore the effectiveness and specific mechanisms of action of framing effects in improving smoking cessation intentions among the general population.

In its latest Global Tobacco Epidemic Report 2025, the World Health Organization (WHO) emphasized graphic health warnings as a cornerstone of global tobacco control initiatives^14^. Although Chinese research has confirmed that well-designed images, particularly pictorial warnings, substantially increase smokers’ quit intentions^15^, China still mandates only text-based warnings on cigarette packs. The absence of pictorial warnings reflects broader challenges in implementing comprehensive tobacco control policies in China. In this context, examining the persuasiveness of gain- and loss-framed cessation messages remains of significant value for China today. Additionally, within China’s pro-smoking cultural milieu, more than 90% of smokers attempt to quit unassisted, rarely accessing evidence-based services such as quitlines or cessation clinics that are widely accepted in Western countries^16,17^. This highlights the distinctive behavioral patterns of Chinese smokers and underscores the limited evidence on whether framing effects observed in international studies are generalizable to the Chinese context. Against this backdrop, we designed two types of texts: one providing information about the benefits of quitting smoking (gain-framing) and the other describing the consequences of smoking (loss-framing). The study sought to assess the immediate associations between these different message frames and quit intentions among adult male smokers in Hangzhou, China, and to examine whether individual psychological characteristics including self-exempt beliefs, self-efficacy, and nicotine dependence, are associated with variations in these associations.

## METHODS

### Study design and participants

A pretest–posttest quasi-experimental study was conducted to evaluate the immediate effects of gain- versus loss-framed cessation messages on quit intentions among adult male smokers in Hangzhou, China. Data collection took place from March to June 2023. Because smoking prevalence in China is predominantly among males^18^, the study population comprised adult male smokers only (age ≥18 years). The urban area of Hangzhou was stratified by distance from the city center into three geographical areas (proximal = Gongshu, intermediate = Binjiang, distal = Fuyang). Within each stratum, fieldwork was conducted in public areas such as community centers or parks. Participants who were approached on site were screened for eligibility (current adult male smoker) and invited to provide verbal consent prior to study participation. For operational feasibility and to minimize contamination between message versions during on-street recruitment, intervention materials were randomly pre-assigned at the interviewer × survey-day level according to a 1:1 allocation schedule. That is, an interviewer on a given day carried and delivered only one message frame (gain or loss) to all approached smokers that day. This approach was chosen to reduce implementation errors arising from frequent material switching and to simplify field logistics. Eligible participants were allocated to receive either gain-framed or loss-framed cessation messages after baseline data collection, and their quit intentions were assessed after the intervention. Due to the nature of the intervention, both researchers and participants were aware of the group allocation during the study. Participants with missing data on the primary outcome or key covariates were excluded from the final analysis.

Based on prior framing studies that reported modest absolute differences in quit-intention responses between gain- and loss-framed messages^9,19^, we estimated that a sample size of 600 participants per group would have >90% power to detect a minimal group difference of 8% on the proportion of positive change in quit-intention between the loss- and gain-framed messages (i.e. from 15% to 23%) and allow for a 10% dropout rate. Under these assumptions, we aimed to recruit 1200 participants in total to ensure adequate study power.

### Data collection

After consenting, participants completed a baseline questionnaire, viewed the assigned framed message, and then completed post-intervention assessments immediately. Anonymous data collection was conducted via the ‘QuestionStar’ platform, with questionnaire completion taking approximately 15 minutes. Additionally, to ensure the rigor of data collection, all survey procedures across different geographical areas strictly followed standardized protocols. For participants with visual impairments or reading difficulties, professionally trained research staff provided one-on-one assistance by reading the questionnaire contents and recording their responses, ensuring that these participants could have an equal chance to participate in the study.

### Intervention

Both intervention texts were content-equivalent, describing the physical, mental, and financial consequences of either quitting or continuing to smoke. The only systematic difference between the conditions was the framing: one emphasized those outcomes as gains from quitting (‘gain-framed’), while the other emphasized the same outcomes as losses from not quitting (‘loss-framed’) – a format consistent with the equivalency-framing established in health communication literature (e.g. ‘You will save money by quitting’ vs ‘You will lose money by continuing to smoke’)^9^. All participants first viewed a prefatory statement: ‘Please read the following information carefully. These health messages are derived from rigorous scientific research, are reliable, and have been reviewed by professionals’. This statement was intended to standardize perceived credibility across conditions before message exposure. Following this, participants read the assigned message text and immediately completed the post–intervention measures. The rationale for this design draws on Prospect Theory^6^ and widely acknowledged framing models^20^, which posit that equivalent message content, when framed differently, can produce significantly different attitudinal and behavioral intentions. The full texts of both message versions are available in the Supplementary file.

### Measurement

*Primary outcome*

All participants rated their intention to quit smoking before and after the intervention, using a 5-point Likert scale (1=very willing to 5=very unwilling). The primary outcome was defined as a binary variable using the ‘change value’ calculated as the difference between pre- and post-intervention quit intentions (i.e. pre-intervention score minus post-intervention score). This metric translates into a positive change score indicating an increase in quit intention, a negative score indicating a decrease, and a zero score indicating no change. The primary outcome was recorded as ‘Yes’ if a positive change score was observed, and ‘No’ otherwise.

*Psychological characteristics*

We also measured the participants’ self-efficacy, self-exempt beliefs, and nicotine dependence at baseline, using the following scales: the Self-Efficacy Scale (6 items, adapted from Schwarzer et al.^21^, with an average score >3 indicating high self-efficacy, assigned a value of 1); the Self-Exempt Beliefs Measure (10 items, adapted from Oakes et al.^22^, with an average score >3 indicating high self-exempt beliefs, assigned a value of 1); and the Fagerström test for nicotine dependence (FTND, 6 items, originally developed by Heatherton et al.^23^, with a total score ≥4 indicating high dependence, assigned a value of 1). Subgroup analyses were performed to explore how individual psychological characteristics could moderate the intervention effects on the primary outcome.

*Sociodemographic characteristics*

Other participant characteristics measured at the baseline survey included: age, education level (less than middle school; high school, including technical secondary; university, including technical diploma; or Master’s degree or higher), marital status (single, married, divorced, or widowed), occupation (Operations, Manager and service, Professionals, Retirees, Others), and annual household income (RMB) (≤50000; 50001–100000; 100001–150000; 150001–200000; or >200000). Self-reported health status was measured by the item: ‘What do you think about your health status when compared with your majority of colleagues/classmates?’. For analysis, responses were dichotomized into Good (excellent or good) and Poor (fair, poor, or bad).

### Statistical analysis

All data were entered into a password protected database using Microsoft Excel, and imported into IBM SPSS Statistics (version 25) for final data analysis. Descriptive analyses were first performed to summarize baseline characteristics of study participants receiving gain-framed messages and those receiving loss-framed messages, respectively. Continuous variables were presented as mean and standard deviation (SD) and compared using t-tests, whereas categorical variables were presented as frequencies and percentages and compared using the chi-squared (χ^2^) tests. Quit intentions before and after the intervention were summarized using both 5-point Likert scales and the change score. To assess intervention effects, the primary outcome was compared between the two groups using logistic regression models with and without covariate adjustment. Based on prior literature^22,24,25^, baseline quit intention, self-efficacy, self-exempt beliefs, and nicotine dependence were pre-defined baseline covariates. Additional participant characteristics were included in the model if significant baseline imbalances were observed between the two groups in the statistical tests. Odds ratios (OR) were estimated with 95% confidence intervals (CI). Statistical tests were two-sided at 5% significance level (i.e. p<0.05).

To explore potential heterogeneity in the framing effects across different study populations, further subgroup analyses were conducted according to individual levels of self-efficacy, self-exempt beliefs, and nicotine dependence at baseline. Separate logistic regression models were used within each subgroup of interest to evaluate potential moderating effects on the primary outcome using different framing messages. The consistency of intervention effects among subgroups was tested using an interaction term with the study groups. Finally, as a sensitivity analysis, propensity score matching (PSM) was conducted with 1:1 nearest-neighbor matching without replacement and a caliper of 0.26 to address potential baseline imbalances between groups.

## RESULTS

**Figure 1:**
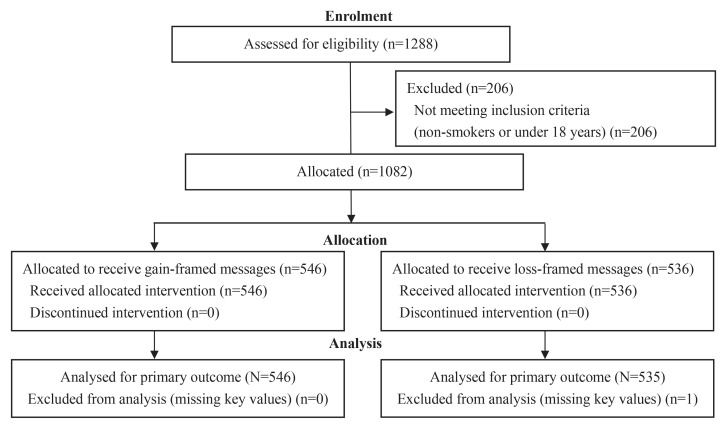
Participant flow diagram for the pretest–posttest quasi-experimental study of gain-framed versus loss-framed cessation text messages, Hangzhou, China, March–June 2023

A total of 1288 individuals were assessed for eligibility in the study. Of these, 206 were excluded because they either did not smoke or were aged <18 years. The remaining 1082 eligible participants were assigned to receive either gain-framed messages (n=546) or loss-framed messages (n=536). All participants received the allocated intervention. One participant with missing data on key variables was excluded from the analysis (Figure 1).

Table 1 presents sociodemographic characteristics of participants at baseline in the two groups. Statistically significant differences were observed in occupation, annual household income, baseline quit intention, self-efficacy, self-exempt beliefs and nicotine dependence (all p<0.05). The mean age was 42.6 ± 15.57 years in the gain-framed group and 41.5 ± 16.43 years in the loss-framed group, showing similar age distributions. In addition, the majority of participants were married, attended a university degree, and reported good self-reported health status.

**Table 1:** Baseline sociodemographic and psychological characteristics of adult male smokers by message framing group, a pretest–posttest quasi-experimental study, Hangzhou, China, March–June 2023 (N=1081)

*Characteristics*	*Gain* *(N=546)* *n (%)*	*Loss* *(N=535)* *n (%)*	*p*
**Age** (years), mean ± SD	42.67 ± 15.57	41.51 ± 16.43	0.235
**Education level**			0.071
Less than middle school	119 (21.8)	145 (27.1)	
High school (including technical secondary)	128 (23.4)	122 (22.8)	
University (including technical diploma)	263 (48.2)	247 (46.2)	
Master’s degree or higher	36 (6.6)	21 (3.9)	
**Marital status**			0.289
Single	157 (28.8)	172 (32.1)	
Married	352 (64.5)	329 (61.5)	
Divorced	24 (4.4)	16 (3.0)	
Widowed	13 (2.4)	18 (3.4)	
**Occupation**			<0.001
Operations	108 (19.8)	142 (26.5)	
Manager and service	192 (35.2)	138 (25.8)	
Professionals	61 (11.2)	29 (5.4)	
Retirees	40 (7.3)	60 (11.2)	
Others	145 (26.6)	166 (31.0)	
**Annual household income** (RMB)			0.031
≤50000	85 (15.6)	63 (11.8)	
50001–100000	134 (24.5)	144 (26.9)	
100001–150000	158 (28.9)	136 (25.4)	
150001–200000	97 (17.8)	90 (16.8)	
>200000	72 (13.2)	102 (19.1)	
**Self-reported health status**			0.312
Good	355 (65.0)	332 (62.1)	
Poor	191 (35.0)	203 (37.9)	
**Baseline quit intention**, mean ± SD	2.57 ± 1.14	2.75 ± 1.12	0.010
Self-exempt beliefs*			<0.001
Low	395 (72.3)	305 (57.0)	
High	151 (27.7)	230 (43.0)	
Self-efficacy*			<0.001
Low	220 (40.3)	151 (28.2)	
High	326 (59.7)	384 (71.8)	
Nicotine dependence*			<0.001
Low	256 (46.9)	333 (62.2)	
High	290 (53.1)	202 (37.8)	

* Self-exempt beliefs and self-efficacy were each categorized as: low (average score ≤3) or high (average score >3); nicotine dependence was categorized as low (total score <4) or high (total score ≥4). Continuous variables are compared using independent-samples t tests; categorical variables are compared using chi-squared (χ^2^) tests. RMB: 1000 Chinese Renminbi about US$140.

Table 2 presents the post-intervention changes in smoking cessation intention, and the unadjusted and adjusted odds ratios (ORs) comparing two groups using logistic regression models. Compared with the gain-framed group (34.6%), a lower proportion of participants in the loss-framed group (30.7%) reported an increase in smoking cessation intention post-intervention with an unadjusted odds ratio of 0.84 (95% CI: 0.65–1.08; p=0.165). After adjusting for occupation, annual household income, baseline quit intention score, self-exempt beliefs, self-efficacy, and nicotine dependence, participants in the loss-framed group had significantly lower odds of reporting increased quit intention (AOR=0.73; 95% CI: 0.55–0.98; p=0.038) compared with those exposed to gain-framed messages.

**Table 2:** Post-intervention change in smoking cessation intention and odds of increased quit intention comparing gain-framed versus loss-framed text messages, pretest–posttest quasi-experimental study, Hangzhou, China, March–June 2023 (N=1081)

	*Post-intervention* *quit intention* *Mean ± SD*	*Positive change in quit intention* post-intervention* *n (%)*		*Unadjusted model*		*Adjusted model*	
		*Yes*	*No*	*OR (95% CI)*	*p*	*AOR (95% CI)*	*p*
**Framing effects**					0.165		0.038
Gain (ref.) (N=546)	2.32 ± 1.10	189 (34.6)	357 (65.4)	1.00		1.00	
Loss (N=535)	2.50 ± 1.11	164 (30.7)	371 (69.3)	0.84 (0.65–1.08)		0.73 (0.55–0.98)	

AOR: adjusted odds ratio. Logistic regression model adjusted for occupation, annual household income, baseline quit intention score, self-exempt beliefs, self-efficacy and nicotine dependence.

* Calculated as baseline score minus post-intervention score; a positive change score (>0) indicates an increase in quit intention post-intervention.

To further illustrate these findings, Table 3 presents quit intentions as categorical variables. Although the baseline distribution of quit intentions was broadly comparable between the two groups, after the intervention, a clearer shift toward stronger intentions to quit was observed in the gain-framed group. Specifically, a higher proportion of participants in the gain-framed group reported being extremely willing (26.9% vs 22.8%) or moderately willing to quit (33.2% vs 27.9%) compared with the loss-framed group.

**Table 3:** Distribution of baseline and post-intervention quit intention levels (5-point Likert scale) by message framing group, pretest–posttest quasi-experimental study, Hangzhou, China, March–June 2023 (N=1081)

	*Gain (N=546)*		*Loss (N=535)*	
	*n*	*%*	*n*	*%*
**Baseline quit intention**				
Extremely willing (1)	106	19.4	80	15.0
Moderately willing (2)	171	31.3	150	28.0
Uncertain (3)	153	28.0	153	28.6
Unwilling (4)	82	15.0	127	23.7
Extremely unwilling (5)	34	6.2	25	4.7
**Post-intervention quit intention**				
Extremely willing (1)	147	26.9	122	22.8
Moderately willing (2)	181	33.2	149	27.9
Uncertain (3)	136	24.9	158	29.5
Unwilling (4)	62	11.4	89	16.6
Extremely unwilling (5)	20	3.7	17	3.2

**Figure 2:**
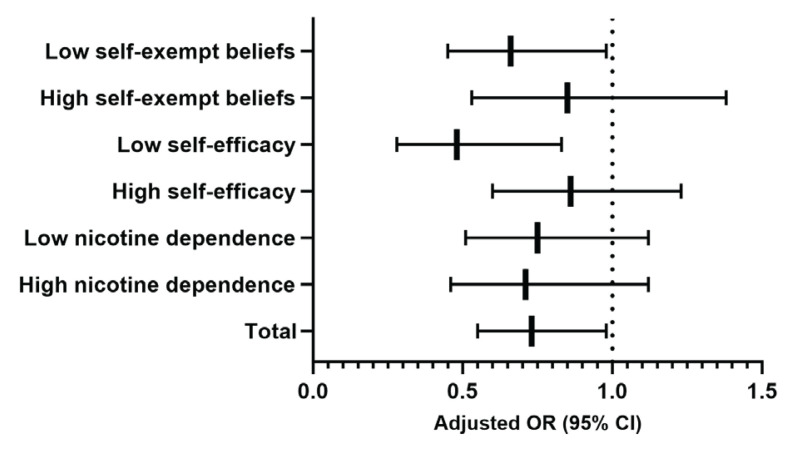
Adjusted odds ratios (AORs) and 95% confidence intervals (CIs) for increased quit intention (primary outcome) comparing loss-framed vs gain-framed (ref.) text messages, overall and by subgroups, pretest–posttest quasi-experimental study, Hangzhou, China, March–June 2023 (N=1081)

The results of subgroup analyses are presented in Table 4. Compared with the gain-framed messages, we found that the loss-framed messages were consistently associated with lower probabilities of reporting increased quit intention in these subgroups (all interaction terms with p>0.1). The differences between two groups were statistically significant among participants with low self-exempt beliefs (AOR=0.66; 95% CI: 0.45–0.96; p=0.031), and low self-efficacy (AOR=0.48; 95% CI: 0.28–0.83; p=0.008). In comparison, no significant differences between framing messages were found in other subgroups. The AORs with corresponding 95% CIs for the overall sample and each subgroup are presented in Figure 2.

**Table 4:** Subgroup analyses: adjusted logistic regression of the association between message framing and increased quit intention stratified by baseline self-exempt beliefs, self-efficacy, and nicotine dependence, pretest–posttest quasi-experimental study, Hangzhou, China, March–June 2023 (N=1081)

	*Framing* *effects*	*Positive change in quit* *intention post-intervention* *n (%)*		*AOR*	*95% CI*	*p*	*p for* *interaction* *effect*
		*Yes*	*No*				
Self-exempt beliefs*							0.702
Low (N=700)	Gain (ref.)	129 (32.7)	266 (67.3)	1.00			
	Loss	89 (29.2)	216 (70.8)	0.66	0.45–0.96	0.031	
High (N=381)	Gain (ref.)	60 (39.7)	91 (60.3)	1.00			
	Loss	75 (32.6)	155 (67.4)	0.85	0.53–1.38	0.515	
Self-efficacy*							0.172
Low (N=371)	Gain (ref.)	74 (33.6)	146 (66.4)	1.00			
	Loss	44 (29.1)	107 (70.9)	0.48	0.28–0.83	0.008	
High (N=710)	Gain (ref.)	115 (35.3)	211 (64.7)	1.00			
	Loss	120 (31.3)	264 (68.7)	0.86	0.60–1.23	0.412	
Nicotine dependence*							0.989
Low (N=589)	Gain (ref.)	95 (37.1)	161 (62.9)	1.00			
	Loss	99 (29.7)	234 (70.3)	0.75	0.51–1.12	0.158	
High (N=492)	Gain (ref.)	94 (32.4)	196 (67.6)	1.00			
	Loss	65 (32.2)	137 (67.8)	0.71	0.46–1.12	0.141	

AOR: adjusted odds ratio. Logistic regression models adjusted for occupation, annual household income, baseline quit intention score, self-exempt beliefs, self-efficacy and/or nicotine dependence as appropriate.

* Self-exempt beliefs and self-efficacy were each categorized as low (average score ≤3) or high (average score >3); nicotine dependence was categorized as low (total score <4) or high (total score ≥4).

### Sensitivity analyses

After PSM matching, covariate balance was achieved (all standardized mean differences <0.1) resulting in 436 matched pairs. Logistic regression models were then re-estimated on the matched sample, including the propensity score as a covariate, with standard errors clustered by matched pair. Results were consistent with the primary adjusted model in Table 2; gain-framed messages remained significantly associated with increased quit intention (AOR=0.75; 95% CI: 0.56–0.99; p=0.041).

## DISCUSSION

Our study examined the adult male smokers in China and explored the influence of different framing messages on their smoking cessation intentions. The study revealed that gain-framed messages tended to be more effective in enhancing adult males’ intentions to quit smoking in China compared to loss-framed messages, particularly in those with low self-exempt beliefs and low self-efficacy. The study finding aligns with theoretical expectations in the health domain that gain-framed messages are more effective in promoting preventive behaviors, including smoking cessation^20^, and is consistent with the majority of existing studies^8-10^. This can be attributed to the fact that smoking cessation is a preventive behavior aimed at maintaining health by mitigating potential health risks^26^. Under this behavioral motivation, gain framing, which emphasizes clear benefits (such as improved health), resonates more strongly with individuals’ cognitive preference for positive outcomes and thus exerts a greater influence compared to loss-framed messages^20^. Furthermore, Croyle and Ditto^27^ emphasized that individuals often exhibit various forms of denial as their initial response when confronted with threatening information. Specifically, smokers tend to develop psychological defense mechanisms against messages highlighting the harms of smoking. A study among Korean adolescents further suggested that individuals with higher levels of psychological resistance exhibited lower smoking cessation intentions when exposed to cigarette warning images^28^. Therefore, directly presenting the negative consequences through loss framing may trigger cognitive resistance, thereby negatively impacting the enhancement of their willingness to quit smoking. Moreover, as highlighted by So^29^, loss-framed messages, due to their overfamiliarity, tend to induce greater information fatigue compared to gain-framed messages. Such fatigue exerts a counterproductive effect on efforts to enhance smoking cessation intentions.

Additionally, given that this study focused on male smokers in China, the persuasive efficacy of gain framing may be particularly pronounced in this population, potentially influenced by cultural and contextual factors. In traditional Chinese culture, there is a deeply ingrained cultural taboo against negative information, particularly topics related to illness and death, which often evoke fear and avoidance^30^. This cultural norm is reflected in the widespread use of euphemistic expressions to address such sensitive subjects in daily life^30^. As a result, this population may exhibit a distinct aversion to loss-framed messages, as it necessitates direct engagement with negative information, which contradicts their cultural predispositions.

Another important factor is that, influenced by the tradition of collectivism, China’s value system places greater emphasis on collective interests^31^. At the same time, in Chinese society, men are often seen as the primary breadwinners of the family and are expected to shoulder significant family responsibilities^32^, reflecting traditional social expectations of adult men’s roles in supporting family and social functions. Gain framing aligns with the positive identity aspirations of Chinese men, emphasizing specific positive outcomes associated with smoking cessation, such as improved health and financial well-being, enhanced protection of family health, and elevated social image. Therefore, gain-framed messages align with male smokers’ personal aspirations and culturally valued outcomes, including the well-being of their families and the broader collective. In contrast, the emphasis on harm and loss in loss framing may cause adult male smokers to feel criticized for their behavior, damaging their dignified image in their family and social roles and causing them to resist. Overall, gain-framed messages align more closely with the deeply ingrained cultural psychology of male smokers in China, and thus may be associated with enhancing their overall willingness to quit smoking.

Further stratified analysis revealed that the relative effectiveness of gain-framed versus loss-framed messages may vary across subgroups. Although all interaction terms were non-significant, these exploratory findings may still provide useful insights into specific populations. Specifically, gain-framed messages tended to be more effective among smokers with low self-exempt beliefs or low self-efficacy. This finding aligns with Mays et al.^24^ who reported that when smoking warnings are delivered through both text and imagery, gain-framed messages are particularly effective for smokers with low self-efficacy. Although these two subgroups differ in characteristics, they share important psychological features: individuals with low self-exempt beliefs cognitively acknowledge the harms of smoking and perceive its consequences as unavoidable, whereas those with low self-efficacy lack confidence in their ability to quit. In both cases, exposure to highly threatening loss-framed messages may trigger defensive responses^27^. By contrast, gain framing is better suited to address these psychological needs, as it mitigates the negative emotional arousal associated with threat-based messaging. Overall, these findings highlight that the effectiveness of this health information intervention is not determined solely by the message content, but also by the psychological and cognitive characteristics of its target audience.

It is noteworthy that the proportion of participants reporting an increase in quit intentions in both groups was much higher than the initial assumptions used in the sample size calculation. Evidence from previous research indicates that groups from different cultural backgrounds may respond differently to health interventions^33^. Therefore, this difference may stem from the fact that the previous studies used for the sample size calculation were based on populations that differ significantly from Chinese male smokers in terms of cultural background, behavior patterns, or psychological characteristics, which could have led to an underestimation of the response to framed messages among Chinese smokers. Additionally, Luisa and other scholars point out that tobacco control policies are effective in reducing smoking rates^34^, and the gradual strengthening of tobacco control policies and the widespread dissemination of public health education in China in recent years may have further increased participants’ receptivity to intervention messages.

Based on these findings, health communication strategies for smoking cessation may benefit from emphasizing the positive benefits of quitting. In designing promotional and intervention materials, gain-framed messaging appears promising and could be considered to maximize persuasive impact. Concurrently, existing tobacco control communications should be critically reviewed to reduce the overreliance on loss framing – particularly avoiding overly threatening language that may evoke psychological avoidance or resistance in certain subgroups of smokers. Given the complexity of framing effects and the heterogeneity of the smoking population, future research could explore audience segmentation and tailored messaging to enhance intervention effectiveness. Neglecting the diversity in smokers’ cognitive and motivational profiles may result in communication strategies that fail to engage their intended recipients or achieve meaningful behavioral outcomes^35^. With the rapid development of digital technologies, precision targeting in smoking cessation interventions has become increasingly feasible. For instance, Strien-Knippenberg et al.^36^ sought to enhance the efficacy of digital smoking cessation programs by refining the PAS (Personal Advice in Stopping Smoking) intervention model. This involves dynamically adjusting message content to individual characteristics and tailoring information presentation to align with users’ autonomy needs, thereby improving engagement and effectiveness^36^.

### Limitations

This study has several limitations that should be acknowledged. First, it employed a one-time short-text intervention to rapidly assess the impact of framing effects on smoking cessation intentions. While this approach is practical for testing short-term effectiveness, the outcome was limited to immediate, self-reported quit intention rather than actual smoking cessation behavior. As a result, the findings primarily reflect short-term associative effects and may be subject to information bias and misclassification; therefore, the results should be interpreted with caution. Moreover, given the quasi-experimental and non-blinded nature of the study, the findings primarily reflect associative rather than definitive causal relationships. Future studies should adopt prospective experimental designs to evaluate the sustained effectiveness of framing effects in real-world settings, including under packaging regulation scenarios.

Second, the study was conducted solely in Hangzhou, a relatively developed city in China. Although we attempted to enhance representativeness by recruiting participants from both central and peripheral districts, the findings may not be generalizable to other regions, especially less-developed areas. Potential geographical clustering at the district level was not explicitly modeled in the analysis, which may have influenced the observed estimates. In addition, although several sociodemographic characteristics were controlled for, residual confounding due to unmeasured factors cannot be completely ruled out. Caution is therefore needed when extrapolating the results to the broader Chinese population. Third, the study specifically targeted male smokers in urban areas. Given the considerable gender differences in smoking prevalence in China, with the male smoking rate among those aged ≥15 years being approximately 24 times that of females^18^, this focus is justified for identifying key intervention populations. Nonetheless, the exclusion of women and rural residents limits the generalizability of the findings, and future research should broaden the scope to include these groups.

## CONCLUSIONS

In this quasi-experimental study among adult male smokers in China, gain-framed text messages were associated with greater immediate effects on increasing quit intention than loss-framed messages. The beneficial effect of gain framing was most apparent among smokers with low self-exempt beliefs and low self-efficacy, suggesting that individual psychological traits may influence responsiveness to message framing. These results suggest that concise gain-framed text messages may be promising, but additional studies using diverse research designs are needed to obtain sufficient evidence regarding their effectiveness and cultural appropriateness among Chinese men.

## Supplementary Material



## Data Availability

The datasets generated and analyzed during the current study are not publicly available due to ethical restrictions related to participant privacy, but de-identified data may be made available from the corresponding author upon reasonable request.
