# Supplementary material for: Framing effects on smoking cessation intentions: A quasi-experimental study of gain- versus loss-framed text messages among male smokers in China
Source: Tob Induc Dis. 2026 Feb 17;24:10.18332/tid/216244. doi: 10.18332/tid/216244 (PMC12911666; doi:10.18332/tid/216244)
Supplement: Supplementary file 1 [file TID-24-22-s1.pdf]

Appendix: Intervention Materials (Content-Equivalent Gain- and Loss-Framed Messages)

The two versions contained equivalent informational content regarding the effects of quitting or continuing smoking on physical health, mental health, finances, and social relationships. The only systematic difference between the conditions was the framing: one emphasized these outcomes as gains from quitting ("gain-framed"), while the other emphasized the same outcomes as losses from continuing smoking ("loss-framed").

No.	Gain-Framed Message (English)	Loss-Framed Message (English)	Gain-Framed Message (Chinese)	Loss-Framed Message (Chinese)
1	By using healthier ways to relieve stress and improve your mood, you will regain control over your behaviors and realize that you are fully capable of managing your emotions without relying on smoking. Tobacco itself does not truly help you solve your problems.	Relying on smoking to relieve stress and improve your mood only causes you to lose control over your behavior and forget that you have the full ability to choose healthier ways to manage your emotions. Tobacco itself does not truly help solve your problems.	用更健康的方式释放压力和疏解心情，您将重新掌控对自己行为的控制权，发现您完全有能力不依赖吸烟处理情绪，烟草本身并不能真正帮助您解决问题。	依赖吸烟行为释放压力、疏解心情，只会让您丧失对自己行为的控制权，忘记您完全有能力选择更加健康的方式处理情绪，烟草本身并不能真正帮助您解决问题。
2	By quitting smoking, you will gain health. Within one week, most harmful substances in your body will be greatly reduced; within one month, your heart and lung functions will improve significantly; within one year, your risk of heart disease will be halved. The earlier you quit, the greater the health benefits.	If you continue smoking, your body will suffer severe damage. Within just one week, large amounts of harmful substances will accumulate in your body; after only one month, your heart and lung functions will decline significantly; after only one year, your risk of heart disease will more than double. The longer you smoke, the more severe the health damage.	戒烟，您将收获健康，戒烟一周您体内的绝大多数有害物质将显著减少，戒烟一个月，您的心肺功能将显著提升，戒烟一年您患心脏病的风险将降低一半。越早戒烟健康效益越大。	如果您继续吸烟，您的身体将受到严重损害。短短一周将有大量有害物质在您体内积累；仅吸烟一个月，您的心肺功能将显著下降；仅吸烟一年，您患心脏病的风险将显著升高一倍以上。吸烟越久，健康损害越严重。
3	By quitting smoking, you will gain wealth. Within one year, you will save over 10,000 RMB and significantly reduce your future medical costs caused by smoking-related diseases.	Smoking will cause you to lose a large amount of wealth, with annual tobacco expenses possibly exceeding 10,000 RMB, and it will significantly increase your future medical costs from smoking-related diseases.	戒烟，您将收获财富，戒烟一年您将节省上万元，并显著减少未来因疾病导致的医疗花费。	吸烟，将使您损失大量财富，在烟草上的花费每年可能有上万元，并将显著增加您未来因疾病导致的医疗花费。
4	After quitting, your family will be protected from secondhand smoke, significantly lowering the risk of birth defects, delayed growth in children, lung cancer, oral cancer, respiratory diseases, and cardiovascular diseases.	Smoking will expose your family to secondhand smoke, which significantly increases the risk of birth defects, delayed growth in children, lung cancer, oral cancer, respiratory diseases, and cardiovascular diseases.	戒烟后，您的家人将免受二手烟的侵害，显著降低胎儿畸形、儿童生长发育迟缓，肺癌、口腔癌、及呼吸道、心血管疾病的发病风险。	吸烟将使您的家人受到二手烟的侵害，二手烟会导致胎儿畸形、儿童生长发育迟缓，肺癌、口腔癌、及呼吸道、心血管疾病的发病风险显著升高。

No.	Gain-Framed Message (English)	Loss-Framed Message (English)	Gain-Framed Message (Chinese)	Loss-Framed Message (Chinese)
5	Although there are individual differences, all smokers suffer harm from smoking. Quitting can prevent disease progression, shorten illness duration, and promote recovery from injuries and illnesses.	Although there are individual differences, all smokers suffer harm from smoking. Smoking worsens many diseases, prolongs illness duration, and hinders recovery from injuries and illnesses.	虽然个体间有差异，但都会遭受吸烟的伤害，戒烟能避免多种疾病加重，缩短病程，有利于外伤和疾病康复。	虽然个体间有差异，但都会遭受吸烟的伤害，吸烟会增加多种疾病的严重程度，延长病程，不利于外伤和疾病康复。
6	Successfully quitting will make your work smoother. Your colleagues will not worry about secondhand smoke, and they will have a good impression of your self-discipline and health, enhancing interpersonal interactions and cooperation opportunities.	Continuing to smoke will not make your work smoother. Your colleagues may worry about secondhand smoke and may have a negative impression of your self-discipline and health, reducing interpersonal interactions and cooperation opportunities.	成功戒烟会让你的工作更加顺利，你的工作伙伴不必担心被动吸烟的风险，也会对您的自律和健康状况产生良好印象，促进人际交往和合作机会。	继续吸烟并不会让你的工作更加顺利，你的工作伙伴可能担心被动吸烟的风险，也可能对您的自律和健康状况产生负面印象，影响人际交往和合作机会。

© 2026 Jin J. et al.
